# Acknowledgment of Reviewers

**DOI:** 10.1002/aps3.1019

**Published:** 2018-02-16

The editors are deeply grateful to the following reviewers (members and nonmembers of the Botanical Society of America) who have generously given of their time to review manuscripts submitted to *Applications in Plant Sciences*. The list includes those who reviewed manuscripts from December 21, 2016, to December 31, 2017. If any reviewer's name has been omitted, we apologize, and ask that a note be sent to the Managing Editor so the list can be corrected.

Agrawal, Anurag

Allen, Julia

Back, Kyoungwhan

Ballard, Harvey

Blackmon, Heath

Blazier, Chris

Blischak, Paul

Bonnet, Pierre

Brosi, Berry

Bruns, Tom

Campbell, Lesley

Chambers, Alan

Chambers, Sally

Chaney, Lindsay

Chang, Peter

Cruse‐Sanders, Jennifer

de Vere, Natasha

Deyholos, Michael

Drummond, Emily

Drummond, Francis

Edger, Patrick

Ellwood, Elizabeth

Endresen, Dag

Escribano‐Rocafort, Adrián G.

Estep, Matt

Faivre, Amy

Fine, Paul

Fishbein, Mark

Folk, Ryan

Forrest, Laura

From, Margaret

Gardner, Dale

Goodell, Karen

Grote, Paul

Hamilton, Matthew

Hegeman, Adrian

Heyduk, Karolina

Hodel, Richard

Hufford, Matthew

Jarvis, David

Jinga, Percy

Johnson, Matthew

Jolles, Diana

Kimura, Mitsuhiro

Lagomarsino, Laura

Landis, Jacob

Latvis, Maribeth

Lavoie, Claude

Lavor, Pâmela

Leonard, Joan

Liang, Chengzhen

Lu‐Irving, Patricia

Macklin, James

MacLeod, Norman

Mandel, Jennifer

Marx, Hannah

McDonnell, Angela

Mei, Wenbin

Merritt, Benjamin

Michaels, Helen

Miller, Andrew

Miller, Ian

Moore, Abigail

Moore, Gloria

Morse, Clinton

Mueller, Rebecca

Muellner‐Riehl, Alexandra

Munson, Richard

Musah, Rabi

Nagel, Dawn

Ogutcen, Ezgi

Orbovic, Vladimir

Orli, Sylvia

Panchen, Zoe

Parducci, Laura

Parks, Matthew

Pease, James

Pec, Gregory

Petit, Sophie

Philpott, Megan

Pyle, Richard

Rafferty, Nicole

Ranathunge, Chathurani

Reynolds, Richard

Riccardi, Greg

Richards, Chris

Roulston, T'ai

Ruas, Claudete

Sarangi, Debalin

Schade, Sven

Scherm, Harald

Schneider, Adam

Schori, Melanie

Serrano Serrano, Martha

Sparks, Erin

Stucky, Brian

Sweeney, Patrick

Tank, David

Tepe, Eric

Theroux‐Rancourt, Guillaume

Thompson, Pamela

Tunison, Robert

Uribe‐Convers, Simon

VanBuren, Bob

Vieira, Leila

Vonderheide, Anne

Weitemier, Kevin

Whipple, Clinton

Wilf, Peter

Winkler, Daniel

Woods, Justin

Xiao, Yuguo

Zavada, Michael

Zenil‐Ferguson, Rosana

Zhang, Wenheng

Zweck, Justin

